# Adaptive Noise Reduction for Sound Event Detection Using Subband-Weighted NMF †

**DOI:** 10.3390/s19143206

**Published:** 2019-07-20

**Authors:** Qing Zhou, Zuren Feng, Emmanouil Benetos

**Affiliations:** 1 State Key Laboratory for Manufacturing Systems Engineering, School of Electronic and Information Engineering, Xi’an Jiaotong University, Xi’an 710049, China; 2 School of Electronic Engineering and Computer Science, Queen Mary University of London, London E1 4NS, UK

**Keywords:** sound event detection, non-stationary noise, weighted non-negative matrix factorization, source separation

## Abstract

Sound event detection in real-world environments suffers from the interference of non-stationary and time-varying noise. This paper presents an adaptive noise reduction method for sound event detection based on non-negative matrix factorization (NMF). First, a scheme for noise dictionary learning from the input noisy signal is employed by the technique of robust NMF, which supports adaptation to noise variations. The estimated noise dictionary is used to develop a supervised source separation framework in combination with a pre-trained event dictionary. Second, to improve the separation quality, we extend the basic NMF model to a weighted form, with the aim of varying the relative importance of the different components when separating a target sound event from noise. With properly designed weights, the separation process is forced to rely more on those dominant event components, whereas the noise gets greatly suppressed. The proposed method is evaluated on a dataset of the rare sound event detection task of the DCASE 2017 challenge, and achieves comparable results to the top-ranking system based on convolutional recurrent neural networks (CRNNs). The proposed weighted NMF method shows an excellent noise reduction ability, and achieves an improvement of an F-score by 5%, compared to the unweighted approach.

## 1. Introduction

Sound events such as screams, gunshots, glass breaks, and so on, are often associated with critical or noteworthy situations. The automatic detection and monitoring of these sound events can be of great use for surveillance purposes. Compared to traditional surveillance systems based on video cameras, audio sensors are insensitive to illumination or occlusion, cheaper, and more suitable for privacy. Moreover, some events like gunshots have no evident visual characteristics and are more suited to audio detection. Nowadays, because of these advantages, the audio information has been exploited solely or jointly with video signals in intelligent surveillance systems. It has found applications including, but not limited to, audio surveillance in public places [1], ambient assisted-living for the elderly or the disabled [2], drone sound detection [3], and animal sound surveying or monitoring [4,5].

Sound event detection involves determining the presence/absence of sound events of interest within continuous audio streams. Generally, research on sound event detection methods has been focused on extracting discriminating audio features, and training effective classifiers for distinguishing different sound classes and noise (see [1] and [6] for a complete review). The input audio signal is typically transformed to the time-frequency domain, and is represented by features like the mel-scale spectral energies, or simply, the magnitude spectrogram. Commonly used classifiers include Gaussian mixture models (GMMs), support vector machines (SVMs), artificial neural networks (ANNs), and non-negative matrix factorization (NMF). More recently, deep learning methods, which are receiving increasing interest, have also been studied for sound event detection, if enough training data are available [7]. However, in real-life scenarios, one factor that degrades detection performance is the interference from highly diverse, unpredictable, and non-stationary noise. Most of the existing methods employing a well-trained classifier over the noise training set cannot handle unseen noise, and also lack the adaptation ability to time-varying noise. Performance of these methods can be severely reduced when the test condition does not match that of the training data, caused by either different recording devices or locations. How to reduce noise, and more importantly, how to generalize well to unknown or changing noise conditions remains a great challenge for sound event detection methods, and is also the focus of this work.

In this paper, we propose an adaptive noise reduction method for sound event detection based on NMF. NMF has proven to be a powerful tool for audio source separation because of its flexibility of additive source modeling, and its effectiveness in representing non-stationary signals [8]. Recently, many sound event detection systems using NMF have been published, with promising results [9,10,11]. NMF models the (non-negative) magnitude spectrogram of a signal as a linear combination of a set of basis vectors (also called a dictionary). By representing an input noisy signal with a concatenated dictionary with event and noise bases, sound events of interest can be separated from noise. One key problem of NMF is that it can easily yield inaccurate separation results, especially in complicated acoustic scenes that are highly non-stationary and contain many interfering sounds that may share overlapping components with the target sound class. As discussed in the literature [10,11,12], because of the spectral overlap of different sound sources, which is also reflected in the redundancy of subspaces spanned by dictionaries, the spectra of interfering sounds in the noise may be wrongly decomposed by the event bases, or vice versa. To address this problem, many variants of NMF have been investigated, and most studies try to incorporate more prior information into NMF so as to gain better control of the separation process. These include learning discriminating dictionaries [13,14], adding source-specific constraints [15,16,17], modelling temporal dynamics [18], and so on.

This paper follows a similar research direction as the above studies, and a weighted variant of NMF is proposed so as to improve the separation quality for sound event detection by utilizing the prior information of both the target events and noise. The motivation is that in the conventional NMF framework, different time-frequency entries within the spectrogram are treated equally, which ignores the relative importance of the different components when distinguishing a target event from noise. It is intuitive that those distinct or significant frequency bands of the target event class are crucial for detection, while those overlapped bands between the target event and noise are confusing and thus unreliable. Weighted NMF (WNMF) provides a way to vary the importance of different components by introducing a weight matrix into the basic NMF model [19]. To the best of our knowledge, there is little research on WNMF, and this is the first attempt as using WNMF for sound event detection. WNMF was first proposed for solving an incomplete data matrix by using binary weights [20], and later for learning local representations for images [21,22]. Recently, several studies on audio source separation using WNMF were published with different weighting purposes. Duong et al. [23] used temporal annotations as weights to highlight frames with less active sources, while a particular type of perceptually motivated weight was introduced in the literature [24] to model the loudness perception of the human auditory system. A very similar work to ours is the one in the literature [25], which used NMF weighted by speech presence probability for speech enhancement.

To achieve a high-quality and noise-robust separation, a novel weighting scheme for NMF is developed in this paper. The aim is to vary the importance of the different time-frequency entries so as to force the separation process, to emphasize those event dominant components instead of suppressing noise. Two weighting strategies are derived, namely: frequency-based and temporal-based. The first one is based on subband importance [26], and is used to enhance those dominant frequencies of the event class, and the latter is used to re-weight different frames and to highlight those frames with a higher event presence probability. A noteworthy property of the proposed weighting scheme is its adaptive noise reduction ability. The weights are designed based on the information of the estimated noise and the target event, and they are adjusted frame-by-frame allowing for noise variations.

Moreover, to tackle time-varying noise, this paper employs a supervised NMF framework that combines a pre-trained event dictionary and an online-adapted noise dictionary. For sound event detection, samples of the target event class are usually available, and an event dictionary can be pre-trained and is usually kept fixed during test. Strategies vary when dealing with noise. The conventional way of learning a noise dictionary over its training set, like what was done by the authors of [9], was not considered, as it lacks flexibility under unseen noise. One option that offers adaptability is the semi-supervised approach, in which the noise dictionary is set to unknown and where it is estimated concurrently with other matrices [10]. However, this method has a limited performance, especially under non-stationary noise. This paper adopts the scheme of noise dictionary learning by the technique of robust NMF [27,28,29]. The noise dictionary is estimated from the current input noisy signal, and can directly describe the surrounding noise. The initial version of the method based on unweighted NMF has been presented in the literature [14], which serves as a baseline in this paper. The major contributions and the strengths of the present work are summarized in the following two points:

- WNMF is applied for audio source separation instead of NMF, which introduces a control on different frequencies and time frames of the input mixture signal. Such a control can help to better emphasize certain important components for distinguishing the target sound events from noise, such as the critical subbands of target sounds, and thus improve the separation quality.
- Noise estimation results from the noise dictionary learning step are exploited in developing both the frequency weights and temporal weights. This produces noise-adapted weights so as to fit the WNMF decomposition to time-varying background noise.

The effectiveness of the proposed method for noise reduction is verified in experiments on datasets from the DCASE 2017 challenge [30]. The proposed method is shown to outperform the unweighted approach, and also achieves comparable results with some of the other state-of-the-art methods. The paper is organized as follows. First, Section 2 reviews the basic model of NMF, as well as its weighted formulation and its application to audio source separation. Section 3 then presents the framework of the proposed method, and gives a detailed description of the noise dictionary learning procedures and the weighting strategies. Section 4 contains the experimental results and discussions. Finally, the conclusions are drawn in Section 5.

## 2. NMF and Weighted NMF

### 2.1. NMF

NMF is a matrix decomposition technique used for generating an additive, part-based representation of non-negative data [31]. Given a non-negative data matrix of $V\in {\mathbb{R}}_{+}^{F\times T}$, NMF tries to approximate **V** by the product of a dictionary matrix $W\in {\mathbb{R}}_{+}^{F\times R}$, and an activation matrix $H\in {\mathbb{R}}_{+}^{R\times T}$, that is, V≈WH. Supposing that **V** represents the magnitude spectrogram of an audio signal with *F* frequency bins and *T* time frames, the columns of **W** can be considered as a set of *R* spectral bases, and the corresponding time-varying gains are stored in the columns of **H**. 

To estimate matrices **W** and **H**, an optimization problem is formulated by minimizing the reconstruction error between the input matrix and its approximation under the non-negativity constraint, that is,

(1) $$\begin{array}{l} \underset{W,H}{\min}D(V|WH)=\sum_{f,t}d\left(V(f,t)|{\left[WH\right]}_{ft}\right)\\ \;s.t.\;W(f,r)\geq 0;\;H(r,t)\geq 0 \end{array},$$

where D(⋅|⋅) denotes the cost or distance function, and d(⋅|⋅) is a scalar cost function. In our algorithm, the generalized Kullback-Leibler (KL) divergence [32] is used as the distance measure, which is defined as d(x|y)=xlog(x/y)−x+y for $x,\;y\in {\mathbb{R}}_{+}$. An iterative algorithm for NMF based on multiplicative update rules is proposed by Lee and Seung [31]. The update rules for the matrices are given by the following:

(2) $$W\leftarrow W⊙\left(\frac{V}{WH}H^{T}\right)/\left(1H^{T}\right),$$

(3) $$H\leftarrow H⊙\left(W^{T}\frac{V}{WH}\right)/\left(W^{T}1\right),$$

in which A⊙B and A/B refer to the element-wise multiplication and division of two matrices, respectively. **1** is an *F* × *T*. matrix with all elements equal to 1, and the superscript T means the transposition of a matrix. Once matrices **W** and **H** are initialized with random non-negative values, the multiplicative update rules can preserve their non-negativity during iteration.

Let us consider the problem of separating two sound classes—a target event class and noise. The common assumption for analyzing a noisy signal containing multiple sound sources is the approximate linear additivity of the magnitude or power spectra. In this work, we use the magnitude spectrogram, which has been shown to achieve satisfactory results in the literature. Given the magnitude spectrogram of an input noisy signal **V**, we have $V\approx V_{s}+V_{n}$. In this study, we used the subscript *s* to indicate the target event class, and *n* for the noise. Supposing that prior information of both sound classes is available as in a supervised case, an event dictionary and a noise dictionary can be trained in advance via standard NMF, denoted by $W_{s}\in {\mathbb{R}}_{+}^{F\times R_{s}}$ and $W_{n}\in {\mathbb{R}}_{+}^{F\times R_{n}}$, where $R_{c}$ is the number of bases for each sound source *c* = *s* or *n*. The NMF decomposition for the source separation takes the following form [8]:

(4) $$V\approx WH=\left[W_{s}\;W_{n}\right]\left[\begin{array}{l} H_{s}\\ H_{n} \end{array}\right].$$

The event spectrogram can then be separated from the noise, and can be estimated by only using the event counterpart $W_{s}H_{s}$, or through a Wiener-type filtering for further smoothing, that is,

(5) $${\hat{V}}_{s}\approx \frac{W_{s}H_{s}}{W_{s}H_{s}+W_{n}H_{n}}⊙V,$$

where the product and division are carried out in an element-wise fashion, the same as those in Equation (2).

Similar to Equation (3), multiplicative update rules for the activation matrices in Equation (4) are derived, as follows:

(6) $$H_{c}\leftarrow H_{c}⊙\left(W_{c}^{T}\frac{V}{WH}\right)/\left(W_{c}^{T}1\right)\;c=s,n.$$

Note that in a semi-supervised case where either the event or noise dictionary is not available, the corresponding dictionary matrix needs to be learned concurrently by the following update rule:

(7) $$W_{c}\leftarrow W_{c}⊙\left(\frac{V}{WH}H_{c}^{T}\right)/\left(1H_{c}^{T}\right)\;c=s,n.$$

### 2.2. Weighted NMF

Weighted NMF [19] modifies the basic model in Equation (4) by incorporating a weight matrix $G\in {\mathbb{R}}_{+}^{F\times T}$, and it is formulated as follows

(8) G⊙V≈G⊙(WH).

Note that when **G** is a matrix with all of the elements equal to 1, Equation (8) is identical to the standard NMF. WNMF can be utilized to emphasize the relative importance of the different components in **V**.

WNMF is solved by minimizing the following weighted reconstruction error [22]:

(9) $$D_{weighted}(V|WH;G)=\sum_{f,t}G(f,t)d\left(V(f,t)|{\left[WH\right]}_{ft}\right).$$

An estimation of the matrices can be achieved by a direct extension of the standard multiplicative update rules, as proposed by Mao and Saul [33], that is,

(10) $$H_{c}\leftarrow H_{c}⊙\left(W_{c}^{T}\frac{G⊙V}{WH}\right)/\left(W_{c}^{T}G\right)\;c=s,n,$$

(11) $$W_{c}\leftarrow W_{c}⊙\left(\frac{G⊙V}{WH}H_{c}^{T}\right)/\left(GH_{c}^{T}\right)\;c=s,n.$$

## 3. Proposed Method

**Figure 1:**
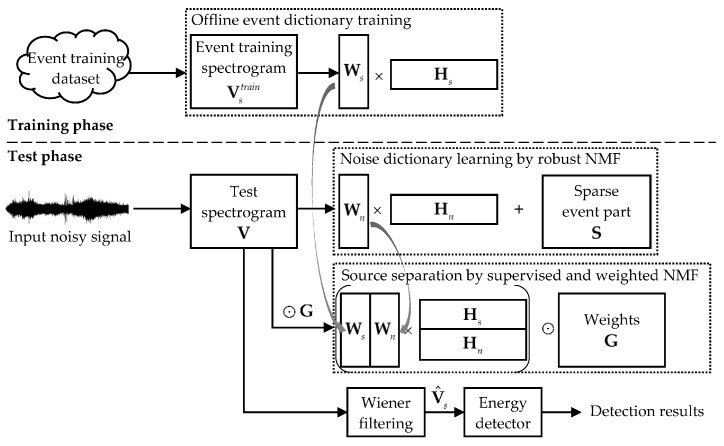
Framework of the proposed sound event detection method based on non-negative matrix factorization (NMF) [14].

This paper proposes a supervised and weighted NMF framework for sound event detection, as shown in Figure 1. The input audio signals are first processed via the short-time Fourier transform (STFT), and magnitude spectrograms are used for audio signal representation. The detection method has two phases—a training phase and a test phase. During training, for each sound event class, an event dictionary is learned using its clean event training data. The spectrograms of all of the event training signals for a specific class are concatenated to yield a data matrix denoted by $V_{s}^{train}\in {\mathbb{R}}_{+}^{F\times T_{train}}$, and the standard NMF is then performed according to Equations (2) and (3). The resulting event dictionary $W_{s}$ is used and kept fixed during the test. In the test phase, the input noisy signal is processed following the three steps of noise dictionary learning, source separation, and event detection. It should be mentioned that the present algorithm is developed in an offline manner, and real-time processing is not emphasized in this paper. For an input test signal, a noise dictionary is estimated from the current input, and then used in the supervised separation process combined with the pre-trained event dictionary. Meanwhile, the time-frequency weights for WNMF are derived according to prior information of the target event class, as well as from the results of the noise estimation. After source separation, the event spectrogram is reconstructed and post-processed by an energy detector so as to generate the detection results. The following subsections will elaborate on the three major steps of the proposed method. 

### 3.1. Noise Dictionary Learning by Robust NMF 

Noise dictionary learning is accomplished by the technique of sparse and low-rank decomposition, also referred to as robust NMF. The underlying idea is to represent a data matrix as the summation of a low-rank matrix and a sparse matrix. Robust NMF has been successfully applied in areas like speech enhancement, and has proved its ability of distinguishing a more regular background from a more variable foreground [28,29]. When applying it to sound event detection, the background noise is usually dense and stable, and can be modeled by the low-rank part. The only assumption for the target sound events is that they happen infrequently and occupy limited entries of the input matrix compared with noise, and thus can be expressed by the sparse part. This is often the case for many audio surveillance applications aimed at monitoring target events like gunshots or screams that rarely happen over a relatively long-time duration.

Given the spectrogram of an input noisy signal **V**, robust NMF decomposes it into the following form:

(12) $$V\approx L_{n}+S=W_{n}H_{n}+S,$$

where $S_{n}\in {\mathbb{R}}_{+}^{F\times T}$ represents the sparse part related to the foreground events, and the low-rank noise part denoted by $L_{n}\in {\mathbb{R}}_{+}^{F\times T}$ is further decomposed via NMF into the product of a noise dictionary and an activation matrix, that is, $L_{n}=W_{n}H_{n}$.

The decomposition in Equation (12) is solved by the following minimization problem [28]:

(13) $$\begin{array}{l} \underset{W_{n},H_{n},S}{\min}D(V|W_{n}H_{n}+S)+\lambda {\left\|S\right\|}_{1}\;\\ \;s.t.\;W(f,r)\geq 0;\;H(r,t)\geq 0;\;S(f,t)\geq 0 \end{array}.$$

The first term of the cost function in Equation (13) is the KL divergence between the input matrix and its approximation. The second term is a sparsity constraint on **S**, which is measured by its *L*_1_-norm, and the parameter *λ* controls the weight of sparsity in the cost function. To estimate the matrices, multiplicative update rules are derived, as follows:

(14) $$W_{n}\leftarrow W_{n}⊙\left(\frac{V}{W_{n}H_{n}+S}H_{n}^{T}\right)/\left(1H_{n}^{T}\right),$$

(15) $$H_{n}\leftarrow H_{n}⊙\left(W_{n}^{T}\frac{V}{W_{n}H_{n}+S}\right)/\left(W_{n}^{T}1\right),$$

(16) $$S\leftarrow S⊙\left(\frac{V}{W_{n}H_{n}+S}\right)/\left(1+\lambda \right).$$

Hence, by solving Equation (13), we can obtain a preliminary separation of the foreground events and noise. The estimated noise dictionary directly models the surrounding noise of the current input, and thus can self-adapt to changing noise conditions. Moreover, the separated low-rank part, that is, $L_{n}$, can be regarded as an estimate of the noise spectrogram, which will be saved for use in the derivation of time-frequency weights in Section 3.2. It should be mentioned that the separated sparse part, that is, S, cannot be directly used for event detection. This is because this decomposition step utilizes no prior information about the target event class, and **S** represents the foreground events of the input and may possibly include other salient undesirable sound events in the background, and thus is not suitable for event detection. The procedure of noise dictionary learning by robust NMF is outlined in Algorithm 1.

**Algorithm 1.** Noise dictionary learning by robust NMF**Input:** spectrogram of an input signal **V**, the number of noise bases $R_{n}$, sparsity parameter λ**Output:** estimated noise dictionary $W_{n}$ and spectrogram $L_{n}$    1:Initialize $W_{n}$, $H_{n}$, and **S** with random non-negative values    2:
**repeat**
    3:    update $W_{n}$, $H_{n}$, and **S** using Equations (14)–(16)    4:**until** convergence    5:Compute $L_{n}=W_{n}H_{n}$

### 3.2. Source Separation by Supervised and Weighted NMF

By combining the pre-trained event dictionary and the estimated noise dictionary, supervised NMF can be implemented for source separation. However, in the conventional model of Equation (4), different frequency bands are treated equally, as well as different time frames, which ignores the relative importance of different components in separating a target event class from noise. To address this problem, we investigate the use of WNMF to quantify the importance of different time-frequency entries within the spectrogram. The intuition is that different frequency bands have a different contribution in constructing a sound source. When detecting a target event from noise, those unique or dominant subbands of the target event play an important role for detection. However, the bands that are shared by these two sound sources can be misleading and result in unstable activations in the NMF model, and thus are unreliable. A good choice would be giving higher weights to those distinct subbands of the target event class. The aim is to force the separation process to put a higher emphasis on those important event-related bands, while the influence of those confusing subbands gets suppressed. In addition, this weighting idea along the frequency axis also applies to the time axis.

Two weighting strategies for WNMF are derived thereby—frequency-based and temporal-based—with the aim to re-weight different frequency bands and time frames, respectively. The former one is based on subband importance and is used to enhance those dominant frequencies of the event class. The latter is used to highlight those frames with a high event presence probability. Furthermore, these two types of weights can be combined to generate time-frequency weights. The formulation of the weights is motivated by the work in [26], which introduced a subband filter that proved to be effective for noise reduction. The proposed subband filter can be regarded as a measure of subband importance, and has the ability of enhancing those significant event subbands while suppressing noise-related bands. Please refer to the literature [26] for detailed derivation and properties of the filter.

#### 3.2.1. Frequency Weighting Based on Subband Importance

The subband weights are computed by making use of the spectral information of both the target event class and the background noise. First, for a specific event class, an averaged spectral template $e_{s}\in {\mathbb{R}}_{+}^{F}$ is extracted according to its clean training data, that is,

(17) $$e_{s}(f)=\frac{1}{T_{train}}\sum_{t}V_{s}^{train}(f,t),$$

in which $V_{s}^{train}\in {\mathbb{R}}_{+}^{F\times T_{train}}$ is the concatenated spectrogram used for event dictionary training.

Likewise, a spectral template of the noise can be computed based on the noise estimation results in Algorithm 1, that is, the estimated noise spectrogram $L_{n}$. In particular, to support adaptation to time-varying noise, a smoothed noise template $e_{n}(t)\in {\mathbb{R}}_{+}^{F}$ is calculated for each frame according to several neighboring frames before and after that frame, that is,

(18) $$e_{n}(f,t)=\frac{1}{number\;of\;averaging\;frames}\sum_{j=\max(1,t-T_{0}/2)}^{\min(T,t+T_{0}/2-1)}L_{n}(f,j).$$

in which the number of averaging frames is equal to $T_{0}$, except for several frames at the beginning and end of $L_{n}$.

Based on Equations (17) and (18), the proposed subband weights are defined as follows:

(19) $$g_{freq}(f,t)=\frac{e_{s}(f)}{e_{n}(f,t)}.$$

**Figure 2:**
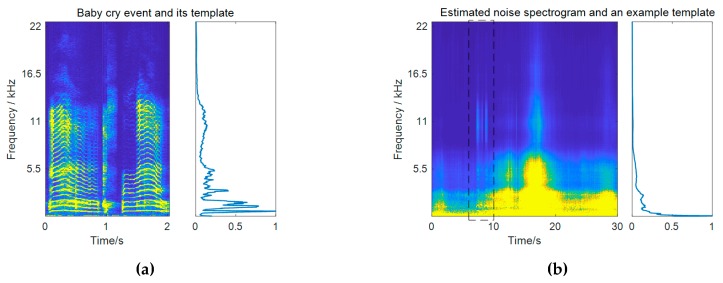

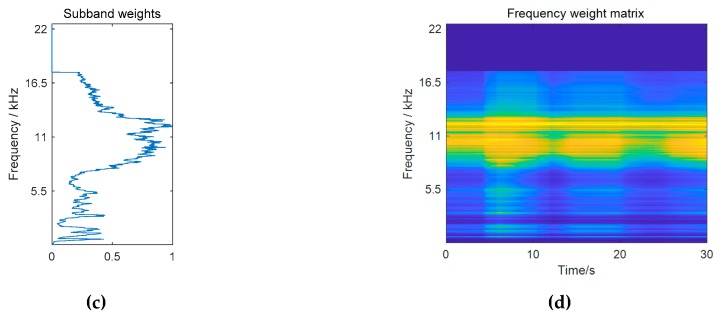
A practical example of calculating subband weights. (**a**) Spectrogram of a baby cry event and its spectral template; (**b**) an example of the estimated noise spectrogram and the noise template for a specific frame (the pictured template is calculated within the frames from 6 s to 10 s, as marked by the dashed box); (**c**) subband weights for that frame; (**d**) subband weight matrix for all frames.

Note that the subband weights are different for each frame according to noise variations. To avoid scaling ambiguity, the subband weights for each frame are normalized to the range between 0 and 1 by dividing them by their maximum value, that is, $g_{freq}(f,t)/\max_{f}\left\{g_{freq}(f,t)\right\}$. By definition, the subband weights measure the ratio of the spectral energy of the target event class to that of the noise in each frequency bin. High weights are given to those bands that are more significant in constructing the target event compared to the noise. More importantly, those confusing bands that have similar importance in constructing the two sources get suppressed. An example of calculating the subband weights for baby cry detection is pictured in Figure 2. It can be observed from the resulting subband weights in Figure 2c that high-frequencies of the baby cry event around 11 kHz get very high weights. Although the baby cry event has strong low-frequency components, these low-frequency regions just get moderate weights, as they are also very strong in the noise. It should be mentioned that in practical calculation, the weights for subbands that have little contributions in constructing the target event have been masked to be a very small value. In the baby cry example, these correspond to frequencies above 17.5 Hz. This operation is necessary in order to avoid meaningless weight values resulting from the division of two very small values.

#### 3.2.2. Temporal Weighting Based on Event Presence Probability

Based on the results in the literature [26], Equation (19) can be regarded as a subband filter in the time-frequency domain. Performing the filtering process on the input noisy spectrogram, we can obtain a denoised spectrogram, defined by the following

(20) $$V_{filtered}(f,t)=g_{freq}(f,t)V(f,t).$$

**Figure 3:**
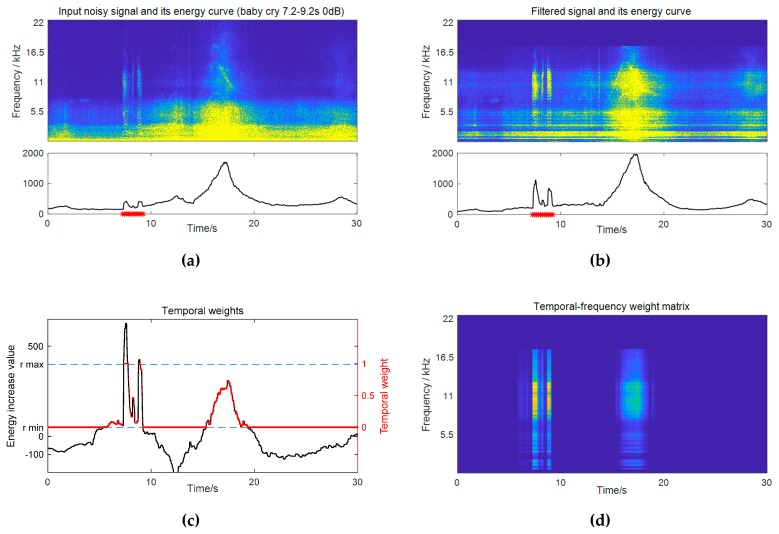
A practical example of calculating temporal weights as well as time-frequency weights. (**a**) Spectrogram and the energy curve of the input noisy signal (frames where the baby cry event is active are marked with *); (**b**) spectrogram and the energy curve of the filtered signal; (**c**) energy increase curve after filtering and the corresponding temporal weights; (**d**) time-frequency weights that combine temporal weights and the subband weights in Figure 2d.

A comparison example between the original and the filtered spectrograms is given in Figure 3a,b, respectively. The test noisy signal is a 30-s recording at a residential area with a baby cry event occurring from 7.2 to 9.2 s at 0 dB. For comparison, the filtered spectrogram is normalized to have the same overall energy as the original input. It can be observed that the high frequencies of the baby cry event get enhanced, whereas the low-frequency noise gets reduced after filtering. 

The effect of this filtering can be further reflected by the energy difference between the filtered signal and the input signal, as shown in Figure 3c. It has been proven in the literature [26] that under certain assumptions, the expectation value of the energy increase values for the frames where a target event is active tend to be greater than those of the noise-only frames. In the presented example, strong interfering sounds exist from 16 to 20 s in the background. After filtering, the frames where the baby cry event is active get the highest energy increase, while the energies of those noise frames get reduced. This energy increase value can be used as a measure for detection, which indicates the probability of a target event, and is calculated as follows:

(21) $$\Delta E(t)=\sum_{f}V_{filtered}(f,t)-\sum_{f}V(f,t).$$

To obtain an estimate of event presence probability, we normalize ΔE(t) to continuous probabilities between 0.01 and 0.99, by two empirically chosen parameters $r_{min}$ and $r_{max}$, that is,

(22) $$g_{temp}(t)=\{\begin{array}{cc} 0.99 & \Delta E(t)\geq r_{max}\\ 0.98\frac{\Delta E(t)-r_{min}}{r_{max}-r_{min}}+0.01 & r_{min}<\Delta E(t)<r_{max}\\ 0.01 & \Delta E(t)\leq r_{min} \end{array}$$

The values of $r_{min}$ and $r_{max}$ are determined based on the results of the subband filtering method, such that $r_{min}$ and $r_{max}$ should guarantee correct detections for frames satisfying $\Delta E(t)\geq r_{max}$ and for frames satisfying $\Delta E(t)\leq r_{min}$, respectively. It was found that our algorithm is not sensitive to the exact values of the parameters. For instance, the parameters are set in the range of $-50\leq r_{min}\leq 100$ and $400\leq r_{max}\leq 600$ for baby cry detection. Note that in the given example, the noise-only frames around 18 s still get moderate weights. This suggests that the accuracy of an event presence probability, that is, temporal weights, depends on the performance of the subband filtering method. A more reliable way is to combine the effects of the subband weights and temporal weights, which yield the following time-frequency weights.

#### 3.2.3. Combined Time-Frequency Weighting

Combining the two types of weights in Equations (19) and (22), we have

(23) $$g_{freq+temp}(f,t)=g_{freq}(f,t)g_{temp}(t),$$

which can be used to re-weight different frequencies and frames concurrently. Choosing one type of the weighting schemes, WNMF-based source separation can be implemented according to Algorithm 2. It should be pointed out that the proposed weights are designed for a specific event class, and the present algorithm is used for single-class event detection.

**Algorithm 2.** Source separation by supervised and weighted NMF**Input:** spectrogram of an input noisy signal **V**,            training spectrogram for the target event class $V_{s}^{train}$ and the event dictionary $W_{s}$,            estimated noise dictionary $W_{n}$ and spectrogram $L_{n}$,            parameters $T_{0}$, $r_{min}$, $r_{max}$, and theTypeOfWeighting**Output:** activations $H_{s}$ and $H_{n}$  1:**switch** theTypeOfWeighting **do**  2:    **case** frequency_weighting  3:        calculate frequency weights using Equations (17)–(19), and set $G(f,t)=g_{freq}(f,t)$  4:    **case** temporal_weighting  5:        calculate temporal weights using Equations (17)–(22) , and set $G(f,t)=g_{temp}(t)$  6:    **case** time_frequency_weighting  7:        calculate time-frequency weights using Equations (17)–(23) , and set $G(f,t)=g_{freq+temp}(f,t)$  8:    **otherwise**  9:        G(f,t)=1, ∀f,t  10:
**endsw**
  11:Initialize $H_{s}$ and $H_{n}$ with random non-negative values  12:
**repeat**
  13:    update $H_{s}$ and $H_{n}$ using Equation (10)  14:**until** convergence

### 3.3. Event Detection

Typically, the detection or classification of NMF-based methods is performed based on the energies of the activations related to the specific class (i.e., $H_{s}$), or taking the activations as new features for a classifier. In this paper, we used the energies of the reconstructed event spectrogram ${\hat{V}}_{s}$ calculated by Equation (5) for event detection, which just can be regarded as a version of smoothed energies of $H_{s}$. For event detection, an energy detector is applied to ${\hat{V}}_{s}$ by thresholding the accumulated energies of all of the frequency bins per frame. High energy values exceeding a threshold in a number of successive frames indicate the presence of a target event. Furthermore, to obtain reliable onset/offset results, necessary post-processing steps are conducted. For example, the output energy sequences are smoothed by median filtering to remove the impulsive values. Very short detected events are removed according to the minimum length of the target event.

## 4. Experimental Results

### 4.1. Dataset and Metric

**Figure 4:**
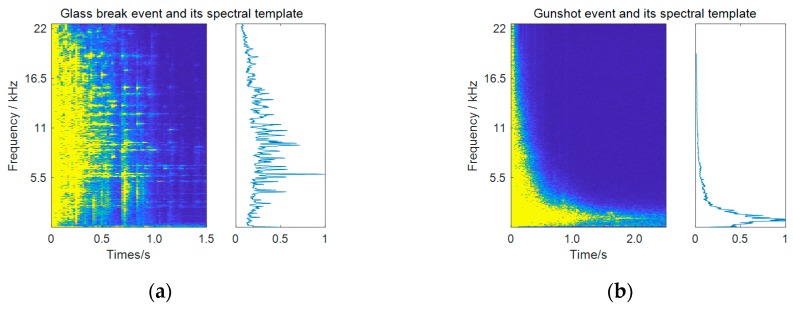
Spectrograms and spectral templates of (**a**) a glass break event and (**b**) a gunshot event.

Experiments were conducted on the dataset of Task 2 of the DCASE 2017 challenge—detection of rare sound events [30]. This task aims to detect three types of sound events (baby cry, glass break, and gunshot) under various environmental noises. Spectrograms of a glass break event and a gunshot event are shown in Figure 4 (see a baby cry event’s in Figure 2a). The audio materials contained isolated clean event samples for each event class (474 unique events in total) and recordings of the background noise (around 9 h). The background noise data were collected from 15 different kinds of real-world environmental scenes, including home, park, metro station, and so on, making it a very complex and diverse noise scenario. This required the detection method in order to be capable of handling different and changing noise conditions. 

For each event class, a mixture of signals of sound events of interest and noise were artificially generated at different SNR levels (6 dB, 0 dB, and −6 dB), and each test recording had a length of 30 s. For the evaluation, the dataset for the test was divided into a development dataset and an evaluation dataset. In each dataset, a total of 500 mixture signals per event class were provided. The audio materials used for creating mixtures of the evaluation set were different from those of the development set. In the experiments, we used the development dataset for choosing the parameters in our algorithm, and reported the final detection results on the evaluation dataset.

It should be mentioned that each sound event class was treated independently in this task, and the problem of recognizing different sound classes was not considered. Detection was done for each event class separately, following the steps in Figure 1. All of the input audio signals were resampled at 44,100 Hz, and the STFT was conducted with a frame length of 40 ms and 50% overlap, which resulted in *F* = 1024 frequency bins. In the training phase, an event dictionary was trained for each event class using its clean event signals. Note that no background audio was used for training in our algorithm. During the test, a noise dictionary was estimated for each 30-s test mixture signal. The parameter $T_{0}$ for calculating the smoothed noise templates in Equation (18) was set to be 200 frames, which corresponded to a 4-s averaging window. To determine the final onset/offset results, the output energy curves were post-processed for smoothing, by a median filter with a length of 11 frames (i.e., 220 ms) for baby cry and glass break classes. However, this procedure was not suitable for the gunshot event, as the gunshot event sounded like an impulse and had a very short duration. The smoothed energy values were then binarized by comparing them to a constant threshold determined for each class. The minimum lengths for rejecting very short detected events were 0.6, 0.2, and 0.2 s for baby cry, glass break, and gunshot classes, respectively.

The evaluation metrics used in the experiments were the event-based error rate and event-based F-score, with a 500 ms onset-only collar [34]. The true positives (TPs), false positives (FPs), and false negatives (FNs) needed to compute the metrics are defined as follows:

- TP: a detected event whose temporal duration overlaps with that of an event in the reference, under the condition that the output onset is within the range of 500 ms of the actual onset;
- FP: a detected event that has no correspondence to any events in the reference under the onset condition;
- FN: an event in the reference that has no correspondence to any events in the system output under the onset condition.

The error rate is defined as follows

(24) $$ER=\frac{FP+FN}{N},$$

where *N* denotes the number of events in the reference. The precision, recall, and F-score are defined as follows

(25) $$P=\frac{TP}{TP+FP},$$

(26) $$R=\frac{TP}{TP+FN},$$

(27) $$F\text{-}score=\frac{2PR}{P+R}.$$

### 4.2. Parameter Selection

The major parameters in our algorithm include the numbers of event and noise bases ($R_{s}$ and $R_{n}$, respectively), and the sparsity parameter *λ* in the robust NMF model for noise dictionary learning. As investigated in our previous work [14], the parameters were set to the optimal ones that obtained the best performance on the development dataset. The search range for each parameter was empirically determined, that is, $R_{c}\in \{16,\;32,\;48,\;64\},\;c=s,n$ and λ∈{0.05, 0.1, 0.2, 0.5, 0.8, 1.0}.

The numbers of the event bases and noise bases had a great effect on the separation quality. For qualitative analysis, using a sufficient number of bases was preferable in order to model the audio sources precisely. However, using too many bases for each audio source may degrade the performance, as it would increase the redundancy of dictionaries and bring the mixing problem, that is, the spectra of the noise may be wrongly described by the event bases or in the reverse way. After a grid search over the pre-defined range, we found that *R_s_* = 32 and *R_n_* = 32 were good choices that would guarantee an excellent performance and also a satisfactory computational load. 

**Figure 5:**
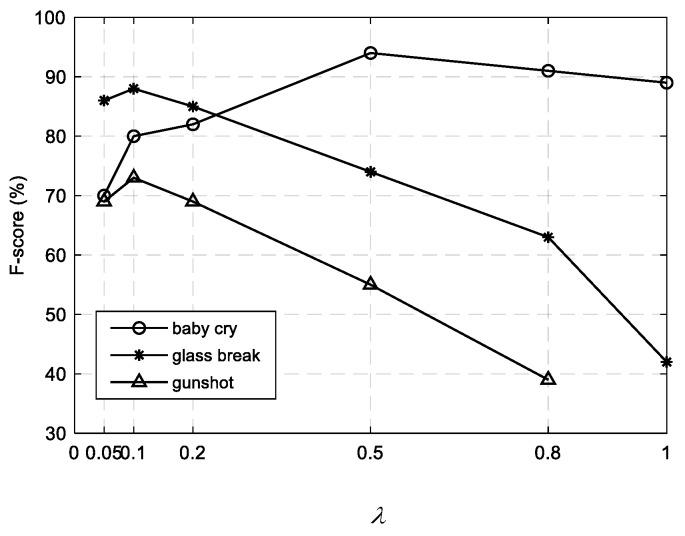
F-score results for three event classes under different values of the sparsity parameter *λ*. The results are obtained on the development dataset by the supervised NMF method without weighting [14].

The sparsity parameter λ used in the noise dictionary learning step controlled the strength of the sparsity constraint on the foreground event part, and thus determined a trade-off between noise reduction and event distortion. A larger λ means a sparser foreground estimate and a more sufficient noise estimate, which may include many contents of foreground events. As the goal of this decomposition is to learn a noise dictionary, it is better not to retain too many event components within the estimated noise part. So, λ should be a relatively small value. According to the F-score results in Figure 5, we found that the best results for the glass break and gunshot classes were achieved at around λ=0.1. The performance degraded under 0.1, and a larger λ also yielded poor results. However, the case for the baby cry class differed a bit, and it turned out that a relatively large λ produced better results. This may be attributed to the considerable difference between the baby cry spectrum and the noise spectrum, as shown in Figure 2a, which enabled a tolerance of the event residue within the noise part. Hence, we set λ=0.5 for the baby cry class and λ=0.1 for the glass breaks and gunshots in the experiments.

### 4.3. Detection Results and Comparative Analysis

To evaluate the proposed method based on the supervised and weighted NMF, we compared its performance with other baseline approaches—the semi-supervised approach that the noise dictionary is not available, as applied in the literature [10], and the supervised approach with the proposed noise estimation, but without weighting [14]. The aim was to separately verify the effectiveness of the proposed two solutions for noise reduction—the noise dictionary learning scheme and the weighting scheme. The error rate and F-score results of the different methods for three event classes are listed in Table 1. The results of the subband filtering method are also provided for reference, as it affects the accuracy of the weights developed in the proposed method. 

**Figure 6:**
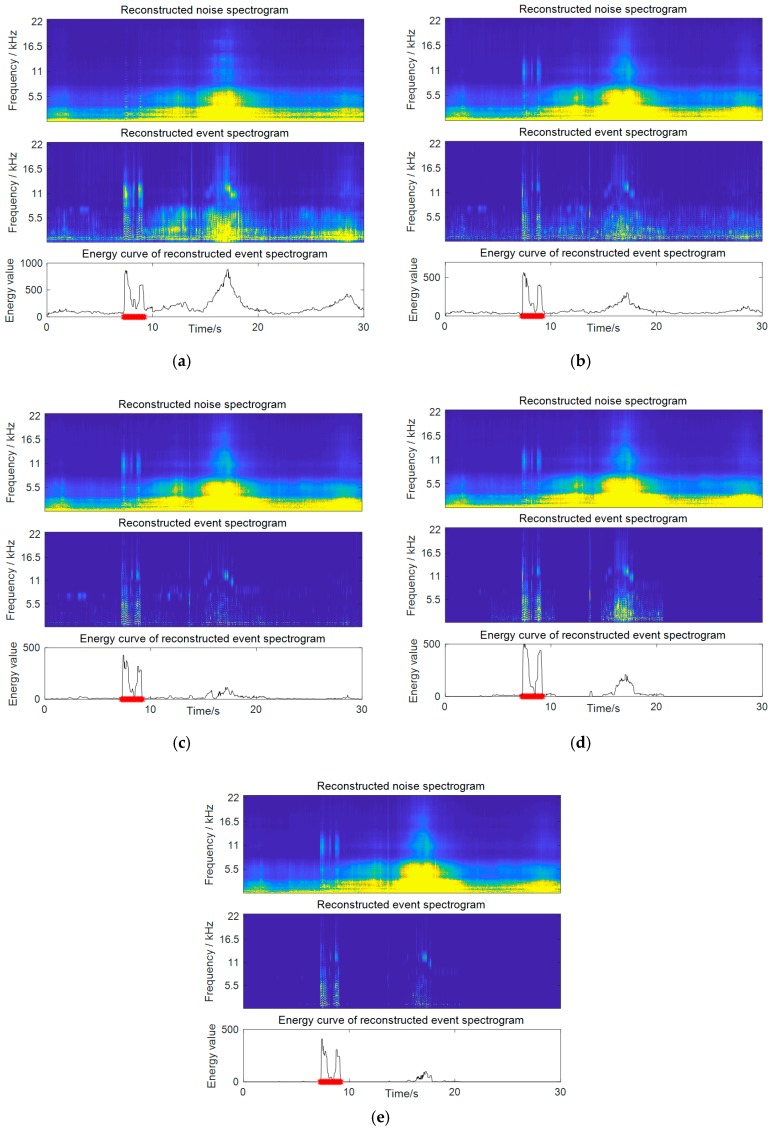
Detection results of the proposed weighted methods compared to two baseline approaches. The test noisy signal is shown in Figure 3a. (**a**) Results of the semi-supervised NMF approach; (**b**) results of the supervised NMF approach with noise dictionary learning, but without weighting. Results of the proposed supervised and weighted NMF approach with (**c**) frequency weighting, (**d**) temporal weighting, and (**e**) time-frequency weighting.

Several observations can be drawn through the results. First, by comparing the supervised and semi-supervised NMF methods (both without weighting), it can be seen that the supervised method outperformed the semi-supervised one with a significant improvement of 8.6% for the F-score. This verifies the effectiveness of the use of an online estimated noise dictionary, which can ensure more accurate separation. A comparison of the separation results on a practical audio example by these two methods is presented in Figure 6a,b, respectively. The test noisy signal is the same as that in Figure 3a. Each picture presents the reconstructed noise and event spectrograms after source separation, as well as the energy curve calculated based on the reconstructed event spectrogram. The supervised method shows a fairly good effect of noise reduction compared with the semi-supervised one, where the strong noise around 18 s in the background gets greatly suppressed. However, the semi-supervised method performed poorly, which is expected, as no prior information about the noise was used in the decomposition. The way of learning a noise dictionary concurrently during separation lacks control over the noise bases, and it may wrongly decompose noise into the event part, or conversely. In the example in Figure 6a, although the spectra of the baby cry event are well captured by the event bases, a large amount of noise spectra are also left in its reconstructed event spectrogram, which leads to incorrect detection.

Second, the proposed weighted NMF methods are superior to the unweighted one, with respect to all three weighting strategies. Among these, the best results were achieved by time-frequency weighting with an average F-sorce of 89.3%, as it made best use of the prior knowledge. The separation results on a practical test signal by three weighting schemes are given in Figure 6c–e, respectively. A further improvement in noise reduction can be observed, compared to the results in Figure 6b, by the unweighted method. It can be seen that time-frequency weighting obtains the cleanest event spectrogram compared to the others, which combines the benefits of both frequency weighting and temporal weighting.

Specifically, the detection results of the frequency weighting and temporal weighting exhibited different characteristics with respect to three event classes, according to Table 1. The performance of the temporal weighting relies on the accuracy of the estimated event presence probability, as indicated by Equation (22). It can be seen that the biggest amount of improvement achieved by temporal weighting was 5.2% for the glass break class, which was also the best performing class in the subband filtering method. As for frequency weighting, it worked well for baby cries and glass breaks, but obtained a limited improvement for gunshots. This can be explained by the spectral similarities between an event class and the noise. The spectra of gunshots were mainly concentrated in low frequencies (as shown in Figure 4b), and thus overlapped with the main frequency region of the ambient noise, whereas the baby cry and glass break classes had many high frequency components and were more distinct to the noise.

**Figure 7:**
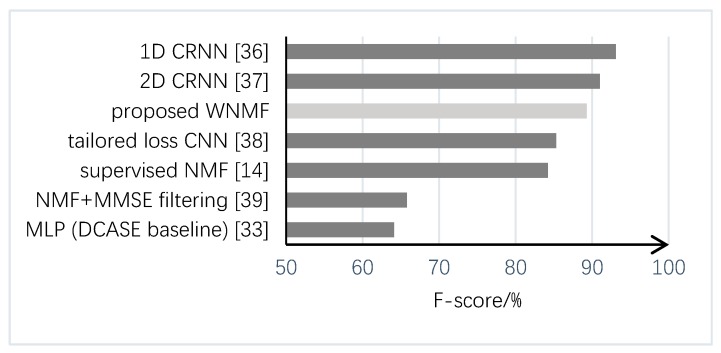
Performance comparison of the proposed method with some other methods submitted to Task 2 of the Detection and Classification of Acoustic Scenes and Events 2017 Workshop (DCASE 2017) challenge.

Finally, we also compared the proposed method with some other state-of-the-art methods submitted to Task 2 of the DCASE 2017 challenge, as shown in Figure 7. The data were taken from the official results of the challenge [35]. It can be seen that the performance of the proposed method (89.3% for F-score) was comparable to those of the top two approaches based on convolutional recurrent neural networks (CRNNs) which obtained F-scores of 93.1% and 91.0%, respectively. The major difference of these two approaches is that the former one applied 1D ConvNet to enable frame-level investigation [36] and the latter used conventional 2D time-frequency spectrograms as input features [37]. Moreover, the proposed method outperforms the method using convolutional neural networks (CNNs) with loss functions tailored for sound event detection [38], and also another NMF-based method which adopted a technique of online noise learning by minimum square error (MMSE) filtering [39]. We argue that the proposed NMF-based method needs less training effort compared with deep learning-based methods, as no background noise data were used for training in our method. It also has the advantage of easy adaptation to new environments, with no need for re-training.

## 5. Conclusions

In this paper, an adaptive noise reduction method based on supervised and weighted NMF is proposed for sound event detection in non-stationary noise. The proposed weighting strategies are guided by both the prior knowledge of sound events and the results from noise estimation, which provide an additional discriminating ability to the original NMF model. For one thing, the weight of each frequency band is quantified as a trade-off between its contributions to constructing the target event class and noise. This forces the NMF decomposition to emphasize those distinct or dominant frequencies of the target event class more. The frequency weighting scheme has shown its effectiveness in improving discrimination when dealing with strong interfering sounds with highly overlapping frequency components. For another, temporal weights based on the event presence probability put higher weights on the decomposition errors of those frames with a higher probability, which helps to produce more accurate separation results. Of all of the weighting schemes, the experimental results show that the best performance is achieved by the combined time-frequency weighting scheme that makes the best use of prior knowledge.

As the proposed method employs a noise estimation technique from the current input noisy signal, which also guides the derivation of both the frequency and temporal weights, the system can be easily adapted to different and time-varying noise conditions. Nevertheless, to ensure performance, the sound events in the training set and the development/evaluation set should better come from the same distribution. In the present algorithm, an average spectral template is extracted for representing a sound event class when determining frequency weights, which has limitations in dealing with the diversity of characteristics within a sound class. Future work will address the adaptation of the proposed approach with multiple templates or templates considering the temporal dynamics of sound events. In addition, another improvement of the present algorithm would be supporting it with real-time processing by using a sliding window, which would make this work more promising for practical use.

## Figures and Tables

**Table 1:** Error rate (ER) and F-score (F) results of the proposed method for three event classes on the evaluation dataset.

Method		Baby Cry		Glass Break		Gunshot		Average	
		ER	F (%)	ER	F (%)	ER	F (%)	ER	F (%)
Proposed supervised NMF +	combined weighting	0.10	94.8	0.06	96.9	0.46	76.2	0.21	89.3
	frequency weighting	0.11	94.0	0.13	93.7	0.51	74.0	0.25	87.2
	temporal weighting	0.14	92.4	0.12	94.3	0.52	73.3	0.26	86.7
	no weighting [14]	0.17	91.4	0.22	89.1	0.55	72.0	0.31	84.2
Semi-supervised NMF		0.29	84.9	0.36	81.3	0.65	60.7	0.43	75.6
Subband filtering [26]		0.62	66.4	0.25	86.7	0.54	67.5	0.47	73.5
